# Length-extension resonator as a force sensor for high-resolution frequency-modulation atomic force microscopy in air

**DOI:** 10.3762/bjnano.7.38

**Published:** 2016-03-15

**Authors:** Hannes Beyer, Tino Wagner, Andreas Stemmer

**Affiliations:** 1 Nanotechnology Group, ETH Zürich, Säumerstrasse 4, 8803 Rüschlikon, Switzerland

**Keywords:** ambient conditions, drift compensation, frequency-modulation atomic force microscopy, high-resolution, length-extension resonator

## Abstract

Frequency-modulation atomic force microscopy has turned into a well-established method to obtain atomic resolution on flat surfaces, but is often limited to ultra-high vacuum conditions and cryogenic temperatures. Measurements under ambient conditions are influenced by variations of the dew point and thin water layers present on practically every surface, complicating stable imaging with high resolution. We demonstrate high-resolution imaging in air using a length-extension resonator operating at small amplitudes. An additional slow feedback compensates for changes in the free resonance frequency, allowing stable imaging over a long period of time with changing environmental conditions.

## Introduction

Frequency-modulated atomic force microscopy (FM-AFM) is the method of choice to image nanoscale structures on surfaces down to the atomic level. Whereas atomic resolution is routinely achieved in ultra-high vacuum (UHV), it remains a challenge under ambient conditions. However, imaging samples in their natural environment down to the atomic level is key to understanding their properties. Several factors such as contamination of the surface, environmental changes, and water layers on the surface hamper high-resolution imaging under ambient conditions. Especially, water layers present on surfaces exposed to air affect the forces acting on the tip, and as a result the stability. Meniscus forces may dominate the interaction and overshadow forces responsible for atomic contrast, namely short-range forces. A viable strategy to circumvent meniscus forces and to achieve atomic resolution is to measure in liquid [1]. Operation with small amplitudes can further help to stay within a single water layer, minimising disturbances which may arise by penetrating several water layers per oscillation [2].

To avoid stability issues such as “jump-to-contact” while working with small amplitudes, sensors with a high stiffness, e.g., short cantilevers, quartz tuning forks, or length-extension resonators are required [3]. In UHV tuning forks have outperformed conventional cantilevers because the high stiffness (*k* ≈ 2 kN/m) of these sensors allows for stable operation at amplitudes down to tens of picometres, thus increasing the sensitivity to short-range forces. In combination with a functionalised tip (e.g., a CO molecule), this ultimately led to the observation of the chemical structure of single molecules [4–5]. Recently, atomic resolution has been achieved with a qPlus sensor in air on potassium bromide and graphite [2,6].

In this paper, we demonstrate the suitability of the piezoelectric self-sensing length-extension resonator (LER) [7–8] for high-resolution FM-AFM imaging in air. The LER has a resonance frequency of about 1 MHz, a Q-factor of approximately 15,000 in air and an effective stiffness of *k*_eff_ = 1.08 MN/m. The effective stiffness amounts to twice the stiffness of a single beam (*k* = 540 kN/m) because the LER consists of two oscillating beams fixed at the center [9]. The very high stiffness allows for operation at very small amplitudes down to tens of picometres and atomic resolution has already been achieved in UHV [10–13]. The sensor is also suited for simultaneous measurements of the frequency shift and tunnelling current [12–14]. Only a few applications of the LER in air or liquid have been reported so far, for example on mica [13,15], Si(111) [16], on a grating [17], HOPG, and DNA origami [18]. Froning et al. [18] also discussed the influence of the environmental conditions on the sensor properties. Temperature and humidity changes lead to variations in resonance frequency and Q-factor, a problem also well-known for regular cantilevers. The problem is aggravated for the LER since the measured signal, i.e., the frequency shift Δ*f*, is small due to the high stiffness of the LER (Δ*f* 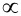 *f*_0_/*k*_eff_). Hence a controlled environment is essential for stable imaging, especially for measurements over a long period of time.

Several approaches have been reported to adjust scanning parameters such that a constant tip–sample distance can be maintained [19–21]. For example, the variation of the amplitude of the second harmonic resonance has been used to adjust the amplitude setpoint of the first harmonic employed for feedback in amplitude-modulated AFM [19]. Another approach is to adjust the topography feedback parameter according to the difference of trace and retrace, which are scanned with different setpoints [20]. Here, we extend the methods reported by Schiener et al. [19] and Fan et al. [21], applying a feedback based on the Q-factor to stabilise the tip–sample distance. In our implementation the ratio of excitation and amplitude of the first harmonic resonance, and thus the Q-factor, is held constant by a slow feedback to compensate for drift of the free resonance frequency.

## Results and Discussion

### Experiment

**Figure 1:**
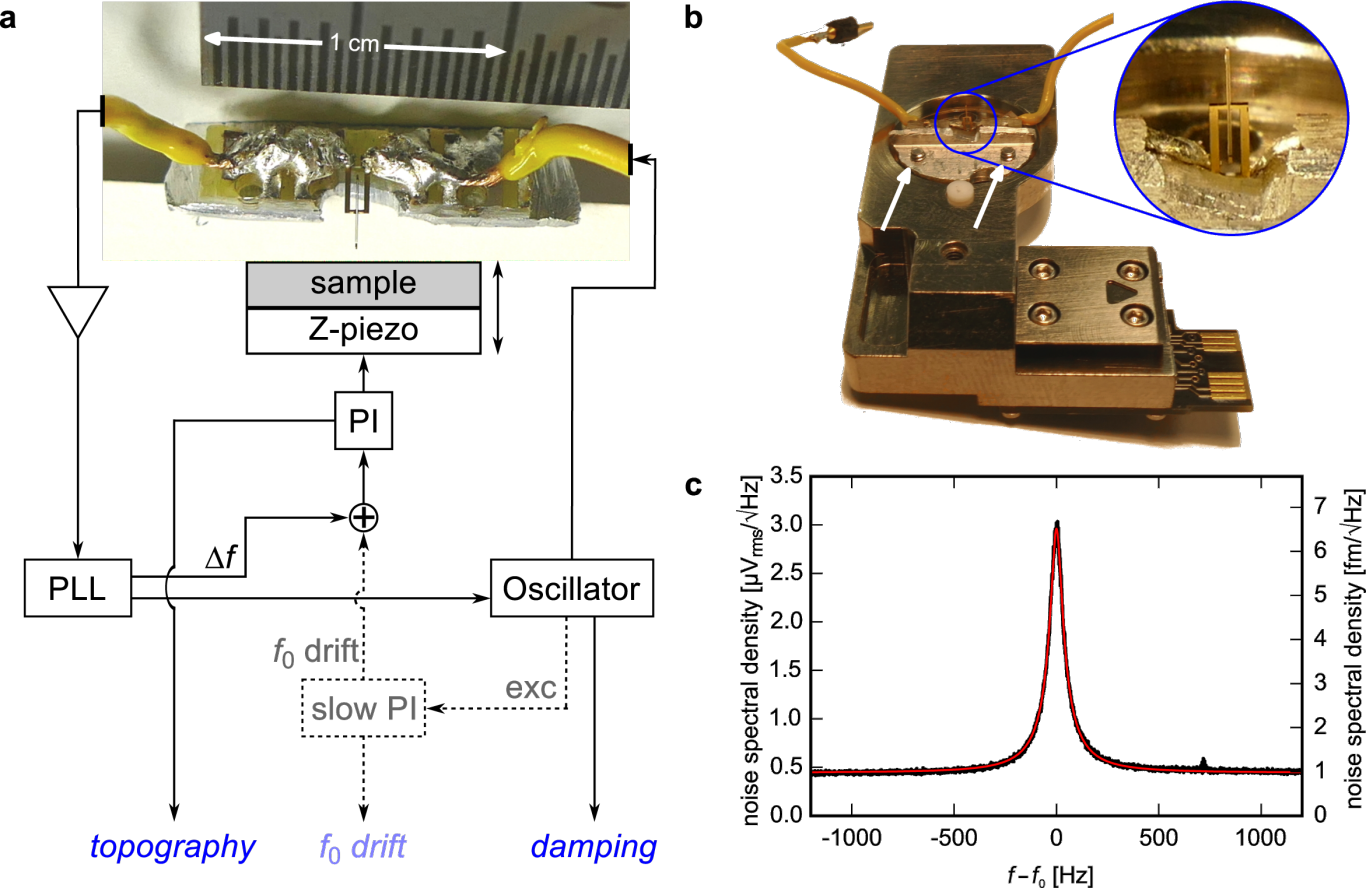
Experimental setup. a) Feedback scheme. The dashed parts enable the slow-drift compensation. Also shown is the LER soldered on a piece of a circuit board, which is glued to an L-shaped metal adapter piece. b) Image of the Cypher droplet holder with LER adapter piece fixed by two screws (white arrows). c) Thermal noise spectrum (black) of a LER with a SSS-NCHR tip attached and a fit of a damped harmonic oscillator (red). The right axis is obtained by multiplying the left axis with the inverse sensitivity 1/*S* = 2.2 nm/V_rms_. Parameters derived from the fit: *Q* = 17,000, *f*_0_ = 999.3 kHz. The detector noise density is 1.0 fm/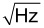.

We use unpackaged length-extension resonators (Microcrystal, Switzerland) and solder both gold electrodes at the base of the sensor to conductive tracks on a piece of a circuit board (Figure 1a). The latter is glued to an L-shaped metal piece, which in turn is screwed to a Cypher droplet holder (Figure 1b) for operation in a Cypher AFM (Asylum Research). The resonator is excited electrically by applying a small AC voltage to one of its electrodes (input) and the displacement-induced piezoelectric current is detected on the other electrode which is connected to a charge amplifier (HQA-15M-10T, FEMTO) (output). Input and output are connected to an oscillator and phased-locked loop (HF2, Zurich Instruments), respectively (see Figure 1a). We use the frequency shift Δ*f* as feedback signal for topography while maintaining a constant amplitude with a separate feedback (constant-amplitude FM-AFM). Tips from commercial cantilevers (e.g., Olympus AC160-R3, Nanosensors SSS-NCH) are glued to the front face of the protruding oscillating beam with silver epoxy (E4110-LV, EPO-TEK Epoxy Technology). Environmental conditions are monitored with a digital temperature and humidity sensor (SHT71, Sensirion AG [22]). Basic image processing (e.g., levelling) is done with the Gwyddion software [23].

To determine the sensitivity *S* of the LER a thermal noise spectrum was acquired around the resonance frequency (Figure 1c). Integration over the noise power spectral density after subtraction of the detector noise floor yields the mean square displacement 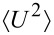 in “V^2^” of the resonator. The sensitivity *S* is then the conversion factor between 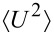 and 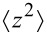 in “nm^2^”: 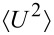 = 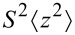. Taking the equipartition theorem, the potential energy of the oscillating beams equals the thermal energy, we can determine *S*:

[1]
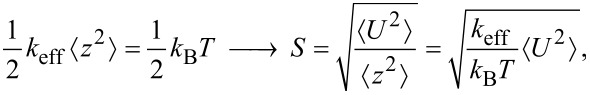


where *k*_eff_ is the effective stiffness, 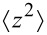 the mean square displacement of the resonator, *k*_B_ the Boltzmann constant and *T* the temperature. The inverse sensitivity amounts to 1/*S* = 2.2 nm/V_rms_. Scaling with 1/*S*, the detector noise density (noise floor in Figure 1c) is 1.0 fm/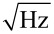, which is comparable to the value measured by Giessibl et al. for signal-to-noise ratio calculations of the LER [9].

### Compensation of environment-induced frequency shift

The frequency shift signal Δ*f* is a measure of the force gradient *k*_ts_ according to Δ*f* = *f*_0_*k*_ts_/2*k*, where *f*_0_ is the free resonance frequency. The high stiffness *k* of the LER leads to a frequency shift signal about 20 times smaller compared to quartz tuning fork sensors. For accurate measurements with the LER it is important to minimise disturbances of the resonance frequency by sources unrelated to the tip–sample interaction.

**Figure 2:**
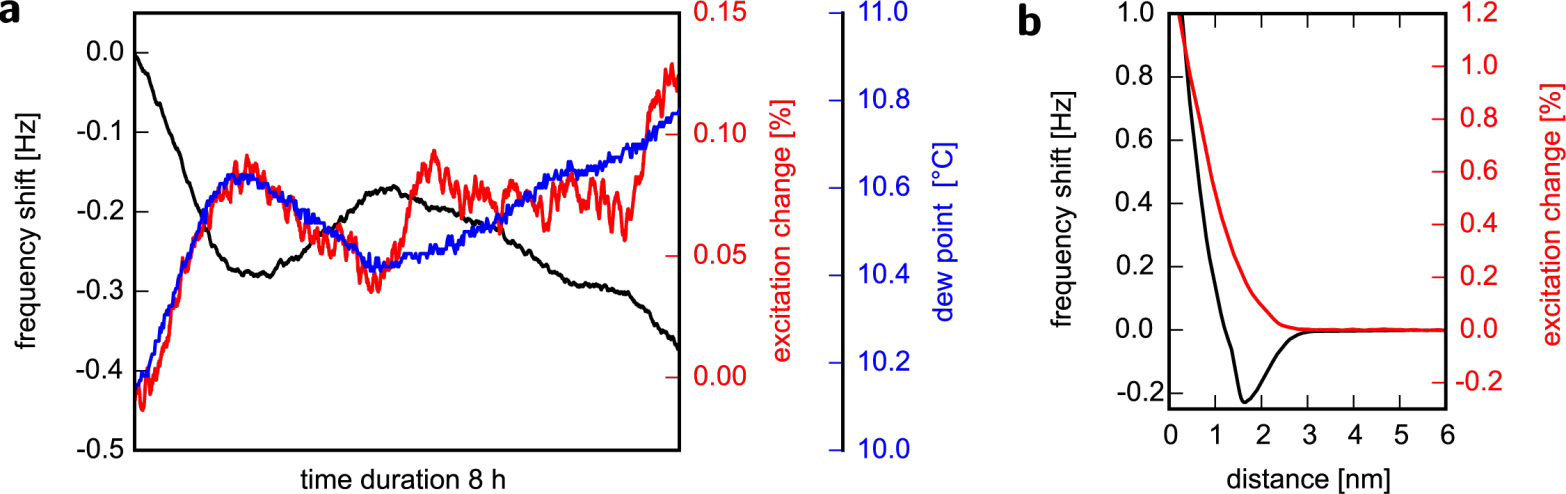
a) Evolution of resonance frequency shift (black), excitation (red), and dew point (blue) over a duration of 8 h. Z-feedback is disabled and the Z-piezo is fully retracted. b) Frequency shift (black) versus distance plot with simultaneously recorded excitation (red) to maintain a constant amplitude of 1.1 nm on a KBr(001) single crystal surface after cleavage in air. The initial excitation is 2.961 mV.

Figure 2a shows the variation of frequency shift, excitation and dew point [22] over time while the sensor is retracted from the surface and Z-feedback is disabled. Frequency shift and damping correlate with environmental conditions. The resonance frequency decreases whereas the damping increases when the dew point rises. Reasons for this behaviour could be, for example, water condensation on the resonator which would add mass, or expansion/contraction of parts of the setup and the solder joints used for mounting the LER. From Figure 2a we find a change in the dew point of about +0.5 K resulting in a change of −0.27 Hz and +0.08% in the resonance frequency and excitation, respectively.

Let us now consider a real measurement at a setpoint of +0.2 Hz. From Figure 2b, an environment-induced shift of the resonance frequency of +0.27 Hz would lead to a change of the tip–sample distance and the excitation of about 300 pm and 0.42%, respectively. This will strongly affect the desired force gradient setpoint and interpretation of data becomes difficult. Furthermore, in a scenario where operation near the frequency shift minimum Δ*f*_min_ is desired, environment-induced drift could cause the setpoint Δ*f*_set_ to cross Δ*f*_min_, leading to retraction (extension) of the Z-piezo if Δ*f*_set_ was originally on the negative (positive) slope branch of the Δ*f*–*z* curve. Again, stable imaging would not be possible.

To overcome such experimental difficulties we have implemented an additional slow feedback to adjust the frequency shift setpoint. The excitation signal is used as input signal of a slow proportional-integral-controller. The setpoint of this slow feedback is determined by the excitation measured at the desired Δ*f* topography setpoint, and thus the desired tip–sample distance. We mainly apply low integrator gain only, resulting in a long time constant (τ ≈ 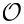 (1 min)), which still allows us to determine damping properties of the sample with the much faster regular amplitude-controller (τ ≈ 5 ms). The slow controller applies an offset to the Δ*f*-signal in order to maintain the excitation setpoint and thus compensates for slow drifts. This is possible because changes of the dew point affect the excitation directly about five times less than the tip–sample distance alteration caused by drift in *f*_0_. Slow drifts of the excitation constitute a source of error of this method. Hence, heterogeneous samples should be orientated such that material properties primarily change along the fast scan axis.

**Figure 3:**
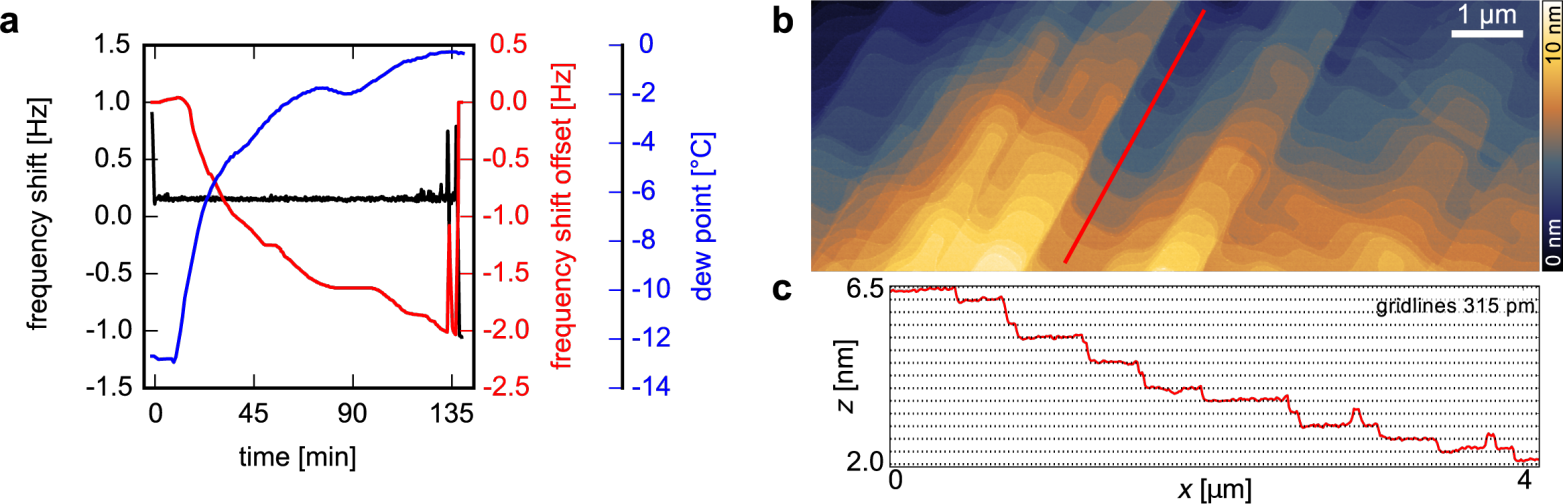
Application of the slow feedback control. a) Evolution of frequency shift Δ*f* (black), frequency shift offset Δ*f*_offset_ (red), and dew point 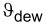 (blue) over 140 min. b) Large scale topography of a KBr surface and c) corresponding height profile along the red line in b). The step height is 315 pm. Scan parameters: *A* = 1.1 nm, Δ*f* = +0.15 Hz, scan speed 10 μm/s.

An example of how this additional slow feedback compensates for environmental changes is shown in Figure 3. Here, consecutive scans over a period of 140 min were performed on a KBr crystal surface with a frequency shift setpoint of +0.15 Hz. The air flow to the AFM housing is controlled via a hose and a reservoir. The air supplied to the reservoir was changed from low humidity air to normal room air after eight minutes. Figure 3a shows dew point 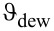, frequency shift Δ*f*, and frequency shift offset Δ*f*_offset_ applied by the slow feedback during the whole duration of the scans. In 140 min the dew point increased by about 12 K. At the beginning (time = 0) the frequency shift drops from Δ*f* = +0.9 Hz to the Δ*f*-setpoint, which is due to piezo engage from the home (retracted) position. During withdrawal of the Z-piezo back to its home position after the scans (time = 138 min), the frequency shift drops to Δ*f* = −1.05 Hz, which results in a total difference of Δ*f*_drift_ = −1.95 Hz attributed to drift. As can be seen from the jump at 133 min (Figure 3a) the tip was retracted before the end of the scans and approached again, most likely due to a bigger contamination on the surface. Note, the frequency shift offset applied for compensation by the slow feedback, Δ*f*_offset_ follows an almost mirrored trace of the dew point, reaching Δ*f*_offset_ = −2.0 Hz just before the end of the scans. This value corresponds very well to the measured Δ*f*_drift_, demonstrating the reliability of the method. In Figure 3b the topography of the last scan is shown together with a height profile along the line indicated (Figure 3c). A typical KBr surface with terraces separated by steps of approximately 315 pm is observed.

### Force regime

**Figure 4:**
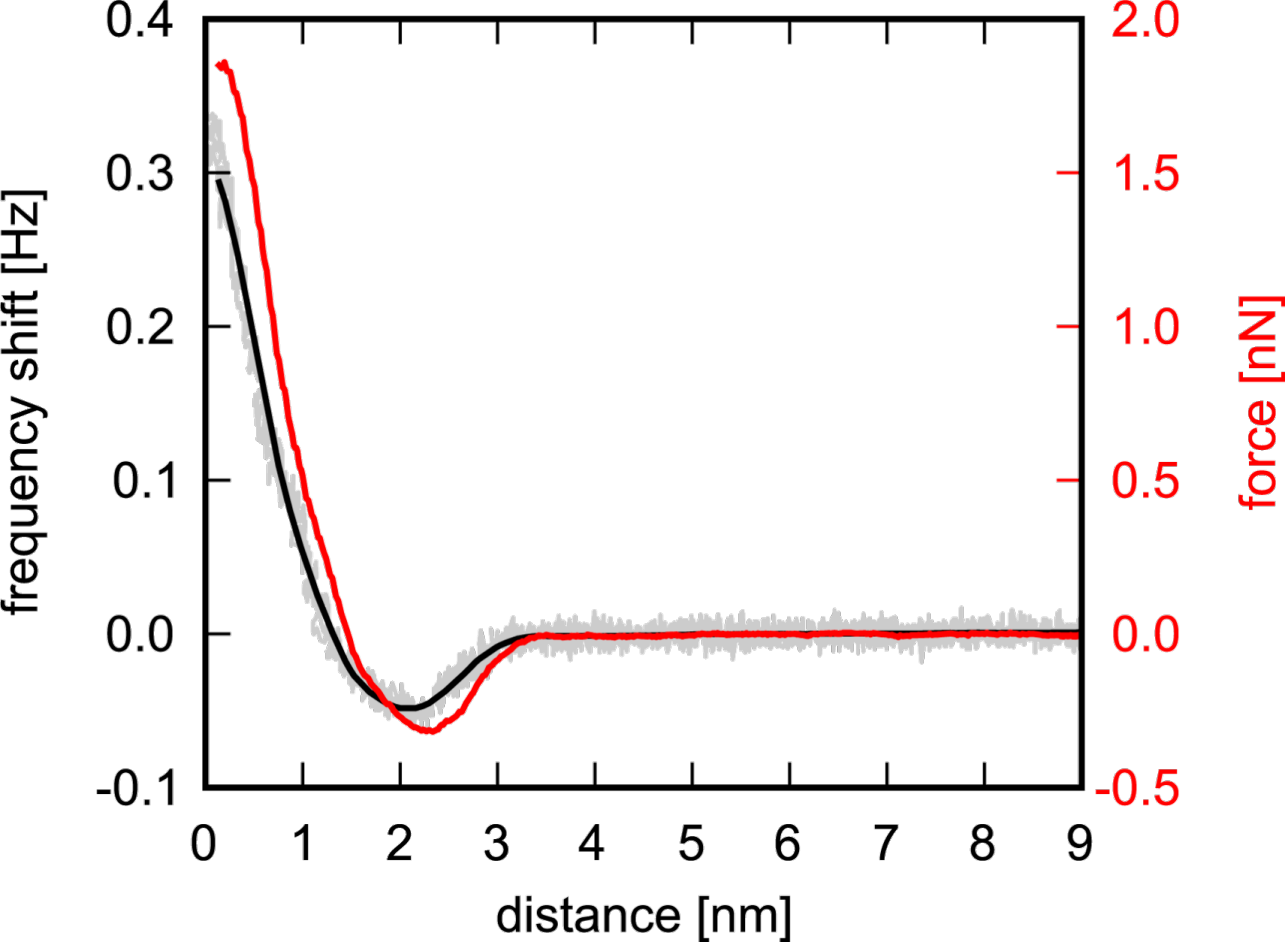
Smoothed frequency-shift (black) versus distance curve on HOPG and tip–sample force *F*_ts_ (red) calculated from the Sader–Jarvis algorithm. The grey curve corresponds to the frequency shift raw data. *A* = 1.1 nm.

As mentioned earlier, the force sensitivity of the LER is lower compared to commercial cantilevers due to the very high stiffness. However, this allows for stable operation with small amplitudes and avoids jump-into-contact. Based on our experience, imaging in the regime of positive slope of Δ*f* often does not provide high resolution whereas imaging on the negative slope is very stable and yields good resolution. The question arises whether non-destructive scanning on delicate samples is still possible in the repulsive regime. To quantify interaction forces we apply the formula derived by Sader and Jarvis [24] to convert the frequency shift into a tip–sample force:

[2]
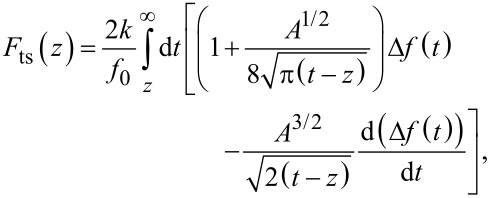


where *f*_0_ is the resonance frequency, *k* the stiffness, *A* the amplitude, and *z* the tip–sample distance. A Δ*f*–*z* curve on HOPG with calculated *F*_ts_ at an amplitude of 1.1 nm is shown in Figure 4. Only a small attractive force regime is present, which can be explained by the high stiffness of the LER. Depending on the sample and its preparation larger attractive forces have also been observed.

**Figure 5:**
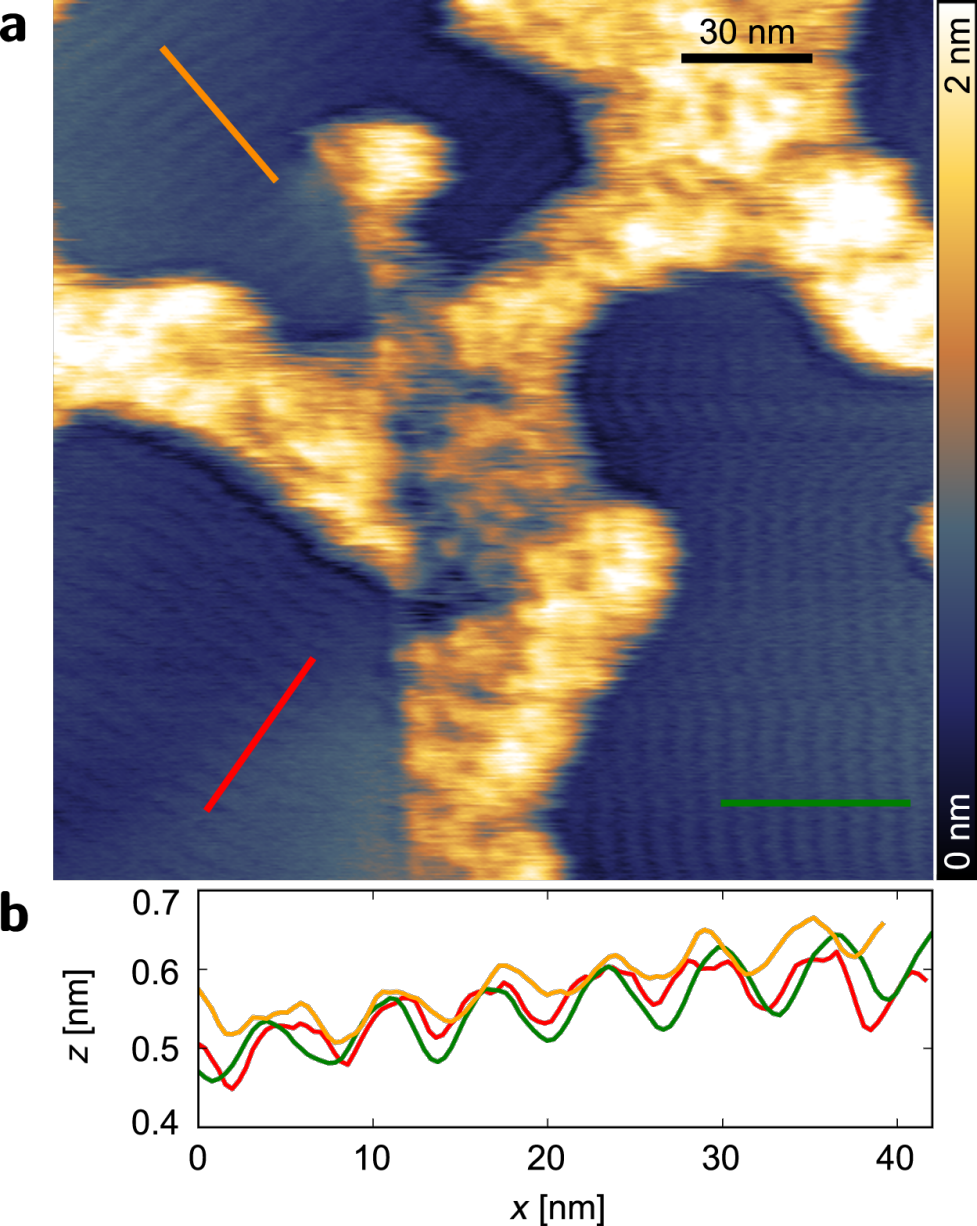
Topography (a) of HOPG after rinsing with Milli-Q water and height profiles (b) along the lines indicated in a) showing the periodic patterns of three domains. *A* = 1.1 nm, Δ*f* = +0.2 Hz, scan speed 977 nm/s.

To prove the feasibility of scanning with small forces a surface decorated by adsorbates was chosen. For this purpose we rinsed a freshly exfoliated (adhesive tape, BT-150E-AT, Nitto Denko) graphite surface with Milli-Q water. It has been reported that in a narrow band of small forces stripes of adsorbed gas molecules can be observed [25]. Indeed, with a setpoint of Δ*f* = +0.2 Hz corresponding to a force of about 1.0 nN three differently orientated domains are observed (Figure 5a). The domains are rotated by an angle of 60° which can be attributed to the underlying hexagonal lattice of graphite. The origin of the stripe pattern is attributed to nitrogen adsorbed through water layers as proposed by Lu et al. [25] from an experiment in a controlled environment. The periodicity of the stripes amounts to 6.2 ± 0.3 nm (Figure 5b). This value differs from the reported 4 nm spacing between the stripes [25–26]. In a later publication Lu et al. also found row spacings of 2 nm for some domains [27], and recently even distances of 6–7 nm have been reported [28–29]. Apparently, several energetically favourable configurations may exist for the adsorption of nitrogen molecules. Further theoretical as well as experimental studies are needed to gain deeper insight into the self-assembly of such molecules on surfaces through water layers.

### Atomic resolution on graphite

**Figure 6:**
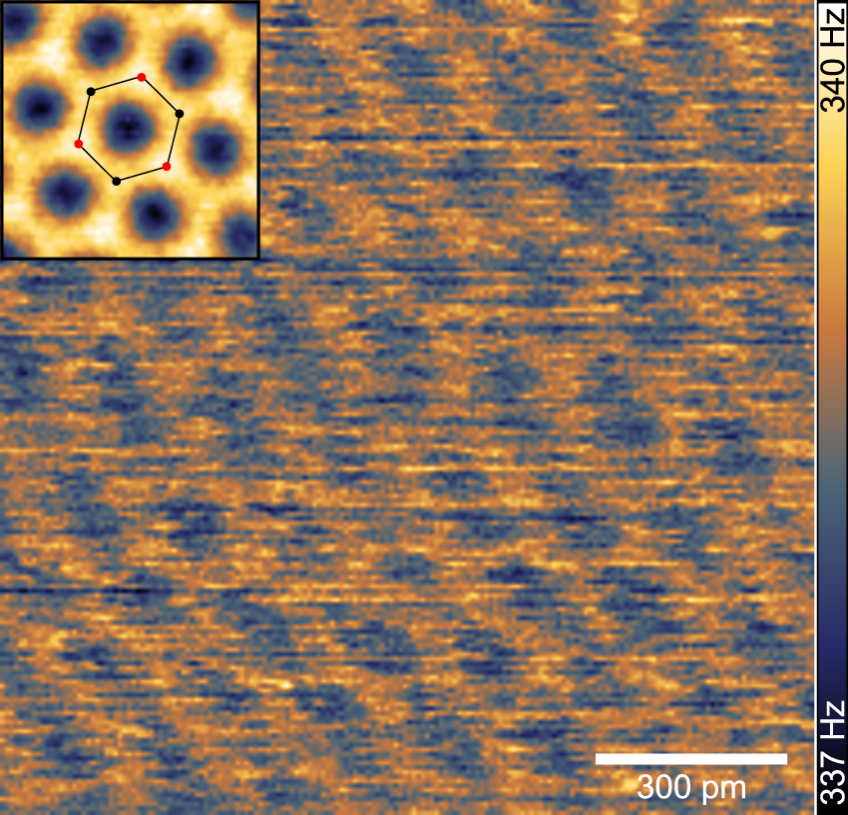
High-resolution detuning image of HOPG in quasi-constant height mode. Inset: 3-fold symmetrised drift-compensated correlation average with overlaid honeycomb structure. *A* = 220 pm, scan speed 58.6 nm/s.

To further demonstrate the high-resolution capability in air, a clean HOPG surface was investigated. The topography feedback gains were set low, resulting in a quasi-constant height mode measurement. Starting from a low positive frequency shift setpoint, the tip–sample distance was gradually decreased until atomic contrast was observed. The hexagonal lattice of the graphite surface appeared between Δ*f* = +315 Hz and +400 Hz. Figure 6 shows a frequency shift image (raw data) acquired with a setpoint of +335 Hz. The raw image is distorted due to drift of the scanner and has been corrected (inset of Figure 6) using a Fourier peak detection method [30]. The drift-corrected image has been processed further by correlation averaging and 3-fold symmetrisation [31]. The honeycomb structure becomes more evident and different repulsive forces for α (above atom in 2nd layer) and β (hollow) sites are observed, too.

Considering the weaker force sensitivity due to the high stiffness of the sensor, high frequency shifts were required to achieve atomic resolution. The interaction forces amount to hundreds of nanonewtons, exceeding the forces observed in contact-mode AFM. Water layers on the surface can contribute substantially to the interaction forces and lead to higher frequency shifts [6,32]. At this stage the atomic contrast obtained at high forces cannot be fully explained yet and further investigations are needed. The operation regime applied here for atomic resolution is rather a “resonant contact” than non-contact mode.

## Conclusion

We have demonstrated high-resolution FM-AFM imaging under ambient conditions with the length-extension resonator. The resonator can be operated stably at small as well as large tip–sample interaction forces. Adsorbates of nitrogen were imaged on HOPG, which paves the road for high-resolution imaging of samples in their natural environment. Furthermore, we have shown atomic resolution imaging on graphite although the interactions are not yet fully understood. A slow feedback maintaining a constant excitation was introduced to compensate for drifts of the free resonance frequency. Stable imaging was demonstrated under extreme variations of the dew point over a period of 140 min. The method could be adapted to other instruments where the Q-factor is rather constant. A modified version could even be used in amplitude-modulated AFM where the average phase signal would be held constant.
